# New horizons in translational bioinformatics: TBC 2013

**DOI:** 10.1186/1755-8794-7-S1-I1

**Published:** 2014-05-08

**Authors:** Ju Han Kim

**Affiliations:** 1 From the Third Annual Translational Bioinformatics Conference (TBC 2013) JW Marriot Hotel, Seoul, Korea; October 2-4, 2013

## Introduction

The Translational Bioinformatics Conference (TBC) has been one of the most successful multidisciplinary conference series in the rapidly emerging field of translational bioinformatics. The Third Annual TBC (TBC 2013) jointly held with the Human Genome Variation meeting (HGV 2013) for 5 days at the JW Marriot Hotel, Seoul, South Korea provided the opportunity to improve the understanding of complex and rare diseases and propose new ways of approaching basic health problems by integrating genomic and clinical data.

There is growing demand for translational bioinformatics approaches that would allow the heterogeneous data from basic research to be integrated systematically into clinical practice in a cohesive and large-scale manner. The expectation is for translational bioinformatics to integrate and correctly extract clinically actionable information from huge data sets at all levels across biological molecules, subcellular compartments, signaling pathways, cells, tissues, organs, and systems. Another challenge is the cultural differences between research scientists and clinicians and their different value systems.

Linking molecular research and clinical applications will enormously benefit human health. Applying translational bioinformatics to next-generation cancer genome sequencing has been very successful in extracting valuable prognostic gene signatures, clinically actionable mutations, novel network biomarkers, and therapeutic targets across many types of malignancy, with examples appearing in areas such as pharmacogenomics, drug developments, and rare Mendelian disorders. I believe that novel translational bioinformatics methodologies developed by multidisciplinary stakeholders will rapidly expand into many clinically disease areas.

## Translational bioinformatics for truly personalized medicine

Personalized medicine involves determining individualized therapies based on the genomic and clinical profiles of specific individuals, which includes utilizing their molecular data. While the predictive power of a plethora of genomic signatures has been successfully demonstrated, dichotomizing phenotypes based on statistical significances is problematic due to the presence of major heterogeneity. In an attempt to realize truly personalized diagnostics and prognostics, Gardeux et al. (University of Illinois at Chicago, USA) have proposed a method of mechanism-level analysis applied to single pairs of samples, such as tumor vs. matched control, primary tumor vs. metastases, and before vs. after treatment. Their N-of-1-pathways predictions not only outperform conventional methods such as Gene-set Enrichment Analysis (GSEA) but also identify unique sample/patient mechanisms, which is a requirement for personalized medicine [1].

Patient-derived xenografts in mouse models have been widely used in the testing of new anticancer drugs in preclinical evaluations of experimental therapeutics due to them representing an approximation to the clinical characteristics of patients. These models involve transplanting human cancer cells into mouse host tissues. However, as Yang et al. (University of Chicago, USA) pointed out [2], profiling the mouse and human transcriptomes separately remains problematic. Yang et al. developed a customized dual-species array (called the H&M array) that they used for cross-species and species-specific hybridization with significantly reduced cross-species hybridization of human and mouse probes, and were able to determine the ratio of stromal to cancer cells based on estimations of the cellularity index of mouse/human mRNA contents in vitro.

## Translational biomarkers and pathophysiological correlates

Biomarkers are indicators of biological states that are useful for the evaluation of physiological states, pathogenic processes, and therapeutic responses. While traditional biomarkers examine the current disease status mainly based on a single molecule, Li et al. (Soochow University, China) focused on the development of phase-specific biomarkers for the progression of prostate cancer through an integrative analysis of gene expression profiling and protein interaction networks [3]. They proposed androgen-receptor nuclear signaling and epidermal-growth-factor-receptor signaling as biomarkers of prostate cancer progression.

Shin and Nam (Ajou University, Korea) have expanded the concept of biomarkers into translational biomarkers [4]. Their predictor-descriptor approach couples together two submodules or biomarkers: a predictor and a descriptor. There is a well-known trade-off when using machine-learning algorithms between prediction performance and the explicit interpretability of the results. While methodologists tend to put more value on the novelty and performance of an algorithm, clinicians are more concerned with how the algorithm obtains a certain level of performance and its real-world usability.

Shin and Nam proposed a model that predicts the survivability of breast cancer patients using a predictor module, and then calculates the variable importance of the prognosis factors using a descriptor module, which also makes it possible to separate patients with similar prognostic profiles. They successfully demonstrated the translational power of the proposed method on the SEER (surveillance, epidemiology, and end results) cancer incidence database [5], which is the most comprehensive source of information on cancer incidence and survival in the USA.

For discriminating disease phenotypes and discovering meaningful biomarkers, Han (Fordam University, USA) proposed applying derivative component analysis (DCA) to high-dimension serum proteomics data [6]. Compared with classic principal- and independent-component analyses, which view each feature as an indecomposable information unit, DCA examines each feature in a multiresolution approach by seeking its derivatives to capture latent data behavior.

The thousands of genetic variants discovered by genome-wide association studies (GWASs) have been shown to explain only a very small proportion of the underlying genetic variance of complex traits. Gene-gene interaction analysis is expected to unveil a significant proportion of missing heritability information about complex traits. Kwon et al. (Seoul National University, Korea) have developed an entropy-based algorithm, IGENT (Information theory-based GEnome-wide gene-gene iNTeraction) [7], that successfully detected gene-gene interactions underlying bipolar disorder in the Wellcome Trust Case Control Consortium (WTCCC), as well as age-related macular degeneration.

## Translational bioinformatics for complex diseases

Despite the recent advances in GWASs and next-generation sequencing technologies associated with the discovery of thousands of mutations and polymorphisms, the causal relationship between pathophysiological molecular mechanisms and therapeutic responses of complex diseases remains unclear. Public databases and numerous biomedical knowledge resources are invaluable when performing translational bioinformatics research. Prosperi et al. (University of Manchester, UK) have attempted to fill this phenome-genome gap by applying a spectrum of linear and nonlinear machine-learning methods to a large clinical and genomic attribute set involving complex clinical conditions: asthma, wheeze, and eczema [8]. Grover et al. (Deakin University, Australia) have attempted to use existing drugs to treat common complex diseases. They extracted 1,497 candidate genes for the 7 complex diseases in the WTCCC GWAS data using a system for predicting candidate genes, and integrated them with publicly available drug databases such as the Therapeutic Target Database, PharmGKB, and DrugBank to identify potentially novel therapeutics for the complex diseases [9].

## Methods for high-performance translation

Improved bioinformatics tools and methods are required for successful translational research. Advanced solutions have been introduced for well-known bioinformatics problems. Wang et al. (University of California, San Diego, USA) have developed a graphics-processing-unit-accelerated massively parallel computing algorithm for miRNA target identification that was 166 times faster than previous methods [10]. Joung et al. (Seoul National University, Korea) detected signatures of inversely correlated expression profiles of miRNAs and their targets related to cancer progression [11]. Ren et al. (Ohio State University, USA) have proposed a layered dynamic programming mapping (LDPMap) approach that uses indexing and two layers of dynamic programming techniques to efficiently map each biomedical term onto a Unified Medical Language System (UMLS) concept [12]. Mapping medical terms onto standardized UMLS concepts is a basic step for biomedical text processing that is hampered by inaccurate query terms. LDPMap is more effective in querying the UMLS Metathesaurus for inaccurately spelled medical terms, long medical terms, and medical terms containing special characters.

The increasing number of multi-institutional collaborations and demand for data integration makes the detection and resolution of measurement-unit conflicts challenging. Samadian et al. (University of British Columbia, Canada) utilized existing ontologies and standards for scientific data representation to build a Semantic Web Service-based approach to automatic measurement-unit harmonization [13]. The translated product of translational bioinformatics may be implemented and carefully evaluated as a clinical decision support system in a hospital information system setting. Lin et al. (National Taiwan University, Taiwan) have implemented and evaluated an automated cellular-phone-based critical-laboratory-value text-alert system for warfarin therapy at a 2,500-bed tertiary teaching hospital in Taiwan [14]. Ji et al. (UC San Diego, USA) proposed a logistic regression-based method for differential privacy against attackers who have auxiliary information based on both private and public datasets [15]. The identification of increasing numbers of valid biomarkers for clinical diagnostics and therapeutics will lead to more products of translational bioinformatics being tightly coupled with clinical decision support systems, which will in turn provide valuable clinical observations on patients' functional states and clinician behaviors, thereby further advancing translational bioinformatics efforts.

The meeting, TBC 2013, has provided the opportunity for translational bioinformatics researchers to come together and substantially improve the understanding of biomolecular and pathophysiological mechanisms, which will contribute greatly to the development of truly personalized diagnostics, prognostics, and therapeutics. I congratulate the speakers and authors at this conference who are shaping the future of how biomedical informatics translates into better clinical practice. Many health topics are increasingly within the scope of translational bioinformatics, including rare and complex human diseases, cancer, biomarkers, pharmacogenomics, drug repositioning, and clinical decision support systems. As Alan Kay said, the best way to predict the future of translational bioinformatics is to invent it.

## Competing interests

The authors declare that they have no competing interests.

## Declarations

This research was supported by a grant of the Korean Health Technology R&D Project, Ministry of Health and Welfare (HI13C2164). Funding for TBC 2013 and open access charge were supported by the Basic Science Research Program through a National Research Foundation of Korea (NRF) grant funded by the Korea government (MSIP) (2013-005540).

This article has been published as part of *BMC Medical Genomics *Volume 7 Supplement 1, 2014: Selected articles from the 3rd Translational Bioinformatics Conference (TBC/ISCB-Asia 2013). The full contents of the supplement are available online at http://www.biomedcentral.com/bmcmedgenomics/supplements/7/S1.
